# Possible Association between Dysfunction of Vitamin D Binding Protein (GC Globulin) and Migraine Attacks

**DOI:** 10.1371/journal.pone.0105319

**Published:** 2014-08-22

**Authors:** Eiichiro Nagata, Natsuko Fujii, Kazuyoshi Hosomichi, Shigeki Mitsunaga, Yoichi Suzuki, Yoichi Mashimo, Hideo Tsukamoto, Tadayuki Satoh, Motoki Osawa, Ituro Inoue, Akira Hata, Shunya Takizawa

**Affiliations:** 1 Department of Neurology, Tokai University School of Medicine, Isehara, Japan; 2 Division of Human Genetics, Department of Integrated Genetics, National Institute of Genetics, Mishima, Japan; 3 Department of Molecular Life Science, Division of Basic Medical Science and Molecular Medicine, Tokai University School of Medicine, Isehara, Japan; 4 Division of Genetic Epidemiology Research Support, Tohoku University School of Medicine, Sendai, Japan; 5 Department of Public Health, Graduate School of Medicine, Chiba University, Chiba, Japan; 6 Educational and Research Center, Tokai University School of Medicine, Isehara, Japan; 7 Department of Forensic Medicine, Tokai University School of Medicine, Isehara, Japan; Odense University hospital, Denmark

## Abstract

To identify the genetic causality of migraine and acute, severe melalgia, we performed a linkage analysis and exome sequencing in a family with four affected individuals. We identified a variant (R21L) in exon 2 of the GC globulin gene, which is involved in the transportation of vitamin D metabolites and acts as a chemotaxic factor; this variant was co-segregated within the family. To investigate the relationship between GC globulin and melalgia, we investigated the cytokine levels in serum samples from the patients and control subjects using a cytokine antibody array. GC globulin can bind to both MCP-1 and RANTES in human serum but has a higher affinity to MCP-1. In cell culture systems, MCP-1 was able to bind to overexpressed wild-type GC globulin but not to the GC globulin variant, and the GC globulin binding affinity to MCP-1 was significantly lower in sera from the patients than in sera from control subjects. A higher concentration of MCP-1 was also observed in sera from the patients. Thus, the dysfunctional GC globulin affected cytokine release, especially the release of MCP-1, and MCP-1 might play important roles in melalgia and migraine.

## Introduction

Migraine is a common, chronic, and incapacitating neurovascular disorder that is characterized by attacks of severe headaches and autonomic nervous system dysfunction [1]–[4].

Migraine is often triggered by stress factors [5], [6], and migraine pain is believed to result from neuronal nociceptive activity in the trigeminal vascular system. Neuropeptides, such as serotonin, calcitonin gene-related peptide (CGRP), and nitric oxide (NO), are released from trigeminal fibers putatively located within the meningeal vasculature, inducing sterile neurogenic inflammation [7]–[9]. Neuroimmune interactions have been increasingly recognized as important elements in nociceptive processing, and recent evidence suggests that the upregulated expression of inflammatory chemotactic cytokines (chemokines) in association with tissue damage or infection may serve not only in the capacity of leukocyte chemotaxis, but also in the generation of hyperexcitable sensory neurons.

In Japan, the overall prevalence of migraine is 8.4% [10]. In migraine patients, a variety of symptoms may precede, accompany, or follow the headache attacks. Notably, a few cases of recurrent limb pain associated with migraine attacks have been reported in children [11].

In this study, we encountered a very rare pedigree that had experienced severe, transient melalgia associated with migraine. We performed exome analyses in this family and identified a non-synonymous variant, R21L, in the *GC* as a candidate.

The possible roles of GC globulin have been evaluated in many biological functions involving the transportation of vitamin D metabolites, and GC globulin has been shown to act as a chemotaxic factor [12]. Accordingly, we also investigated the roles of cytokines in the pathophysiology of melalgia in this family.

## Results

### Genomic regions detected using a linkage analysis

A total of 443,169 single nucleotide polymorphisms (SNPs) were genotyped with Affymetrix annotation; monomorphic SNPs, X-linked SNPs, and SNPs with Mendelian errors were then excluded, leaving 274,743 effective SNPs for the linkage analysis. However, several concerns must be taken into consideration when performing a linkage analysis, as follows: 1) SNPs in pair-wise linkage disequilibrium could inflate the linkage statistics [13], 2) SNP typing errors could lead to inaccurate linkage results, and 3) it might be impossible to process all the SNPs simultaneously because of computational limitations (memory and time required to perform the computations). To overcome these difficulties, we divided the overall data set into 20 subsets by selecting one every 20 successive SNPs. Thus, 9,463 data items were included in each subset, which covered an average interval of 0.32 Mb. Then, we performed a multi-point linkage analysis for each of the 20 subsets and calculated the average LOD scores for all the subsets.

Positive evidence of linkage (LOD score exceeding 1.5) with the highest LOD score (1.74) was observed for the following eight loci: 4p (chr4: 69,806,274–70,004,019), 7q (chr7: 146,671,680–150,707,757), 8q (chr8: 109,132,749–109,621,594 and chr8: 137,637,209–138,489,882), 10p (chr10: 14,017,991–18,589,272 and chr10: 34,309,652–35,120,917), 13q (chr13: 111,798,712–112,053,389) and 18p (chr18: 27,430,719–35,168,576). These eight linkage regions were then used to filter the candidate variants.

### Filtering of candidate variants using a combination of linkage analysis and exome sequencing

We detected high-quality variants satisfying 3 criteria: 1) the SNP quality had to be no less than 20, 2) no less than 20 reads had to support the variant allele, and 3) the SNP could not be located in a segmental duplicated region (over 0.95 similarity). In total, 13,989 exomic variants (including 6,421 non-synonymous and 7,568 synonymous variants) were detected using exome sequencing. We next performed three filtering procedures to narrow down the candidates for the disease-causing variant. The first procedure selected variants based on the linkage results (LOD scores exceeding 1.50). Eight loci with an LOD score of 1.74 (the maximum value in the present study) were first considered, and regions with an LOD score exceeding 1.5 were screened for the causal variant. According to the first filtering step, 347 variants located within regions with a high LOD score were selected. The second filtering step was performed based on the observed allelic frequency: a range of 0.4 to 0.6 was used as the cut-off threshold, since we applied the pooled method and the affected individuals in the family were expected to share the same causal variant in a heterozygous state. Ninety-six of the 347 variants were selected as shared heterozygous variants. The last filtering step was performed based on the variant annotation of the dbSNP131 entry and amino acid changes that would result in novel or very rare variants. Familial melalgia is a rare disease; thus, we assumed that the causal variant should not have been registered in public databases or had been registered with a very low frequency. As a result of these three filtering steps, only 16 of the 96 variants were identified as novel, non-synonymous variants. These 16 variants were found in nine genes as follows: three of the variants were located within chr4: 69,806,274–70,004,019, seven were located within chr7: 146,671,680–150,707,757, three were located within chr10: 34,309,652–35,120,917, two were located within chr8:137,637,209–138,489,882, and one was located within chr18:27,430,719–35,168,576. The familial co-segregation of all 16 candidate variants with the disease status was examined using the Sanger method for all the family members. Only two variants in *GC* and *HTN1* were selected by this filtering method. However, *HTN1*, which is known as salivary protein histatin 1 and is considered to be a major precursor of the protective proteinaceous structure on tooth surfaces, was excluded as a candidate based on its biological function.

Although we identified one gene as the causality of familial malalgia by combining linkage and exome analyses, there might exist an interacting factor, which is very difficult to identify in one family.

### Mutation of GC globulin (vitamin D binding protein)

**Figure 1:**
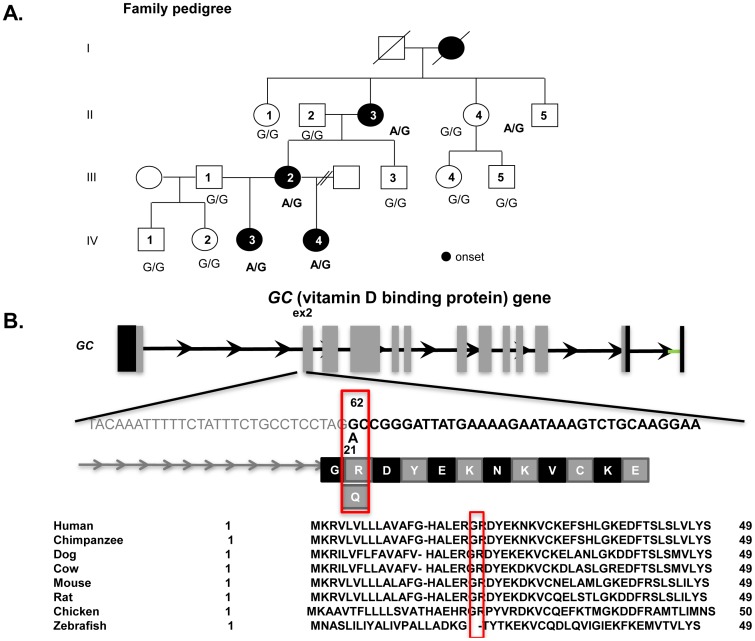
Family pedigree and sequence of *GC* (vitamin D binding protein). **A**) Squares: males; circles: females; solid symbols: patients with severe myalgia and migraine. **B**) The mutation results in a substitution at the 62^nd^ amino acid (G to A).

We eventually identified one novel non-synonymous variant in the *GC* at 14q21 that was co-segregated within the family, except in II-5 (Figure 1). Of note, II-5 had experienced an episode of melalgia when he was 2–3 years old. The variant was observed in 5 out of 238 healthy Japanese individuals (5 out of 476 chromosomes, frequency of 0.005).

The variant led to an amino acid substitution (arginine to leucine) at amino acid position 21 on exon 2, which is the second amino acid position of the mature protein. The variant score of SIFT [14] was 0.01, and the PolyPhen2 [15] damaging score for amino acid substitution was 0.99, resulting in a “probably damaging” status. Although the variant did not correspond to a known domain, it was located in a highly conserved sequence across various vertebrate species. The variant scores of PhyloP [16] and GERP (Genomic Evolutionary Rate Profiling)[17] for conservation were 0.93 and 3.57, respectively.

### Concentrations of 25OH and 1, 25OH as well as CGRP

In serum samples from the patients, the levels of 25OH and 1, 25OH vitamin D were within the normal ranges (Table 1). As for the level of CGRP, no significant difference was observed between the patients and the control subjects (Table 2).

**Table 1:** Serum concentrations of vitamin D (25 (OH) D, and 1, 25 (OH)2 D).

25(OH)D (ng/mL)	III-1	17
(normal range: 9–33.9 ng/mL)	III-2	10.7
	IV-3	39.7
	IV-4	15.7

1,25(OH)2D (pg/mL)	III-1	34
(normal range: 20–60 pg/mL)	III-2	21
	IV-3	41
	IV-4	57

No significant differences in the serum concentrations of vitamin D (25 (OH) D and 1, 25 (OH)_2_ D) were seen between the patients and the healthy control subjects.

III-1: Not patient in this family; P: patients.

**Table 2:** Serum concentrations of CGRP (pg/mL).

Control	86.9±7.2
Patients	86.7±9.5*

No significant difference in the serum CGRP concentration was seen between the patients and the healthy control subjects.

*: no significant difference.

### MCP-1 and RANTES bind to GC globulin

**Figure 2:**
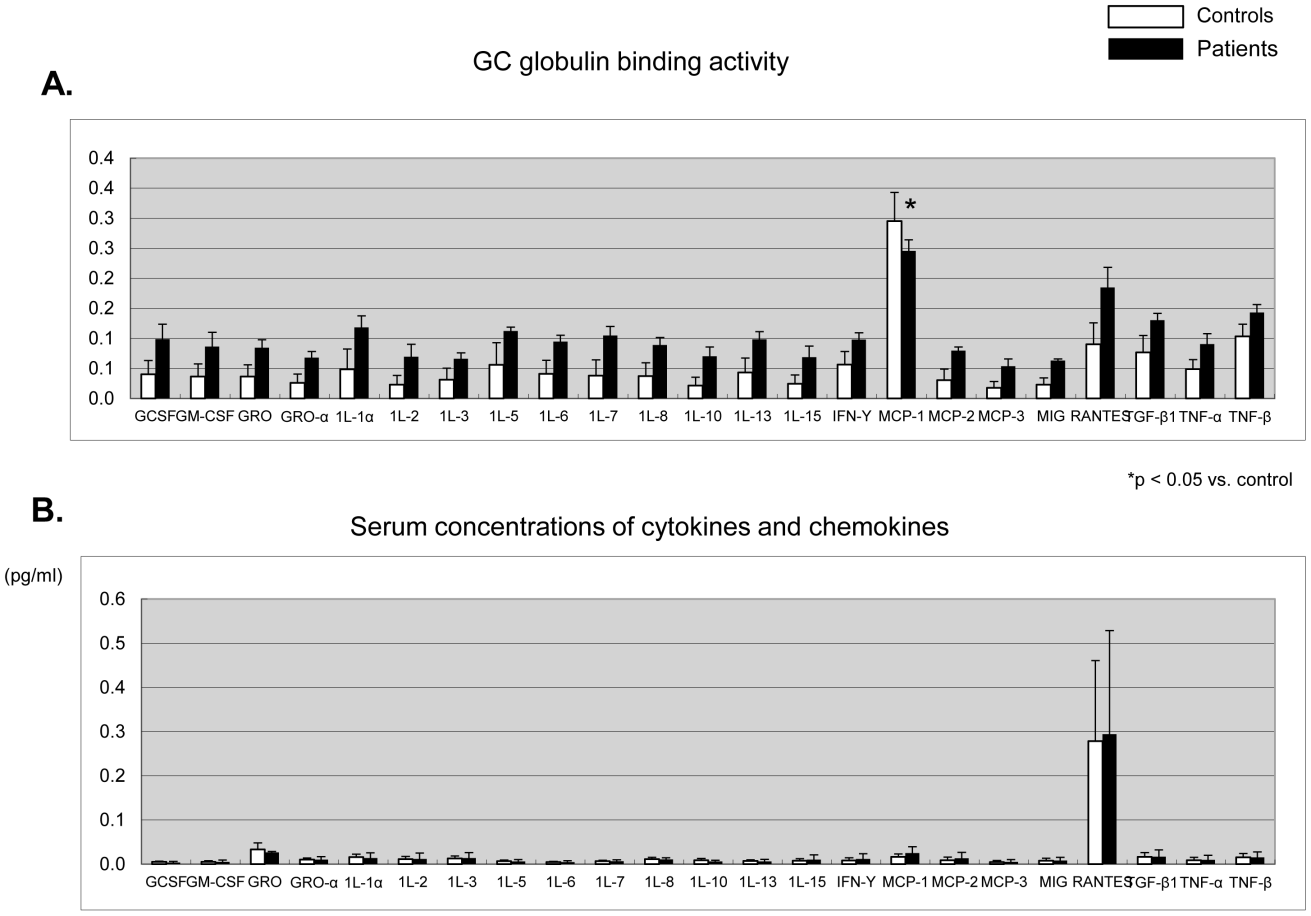
Cytokine antibody array for GC globulin binding activity and serum cytokines. The GC globulin binding affinity to MCP-1 was the highest among all the serum cytokines (A), while RANTES had the highest concentration among the serum cytokines (B). In sera from the patients, the GC globulin binding affinity to MCP-1 was significantly lower than that in sera from the control subjects (A), while the concentration of MCP-1 in sera from the patients tended to be lower than that in sera from the control subjects (B). White bars: healthy control subjects; Black bars: patients with severe myalgia and migraine.

In this study, we found that GC globulin had a high affinity to MCP-1 in human serum (Figure 2A). The serum concentration of RANTES was relatively high among the concentrations of human serum cytokines, although a few other cytokines were present (Figure 2B). No significant difference in the serum concentration of RANTES was seen between the patients and the control subjects.

**Figure 3:**
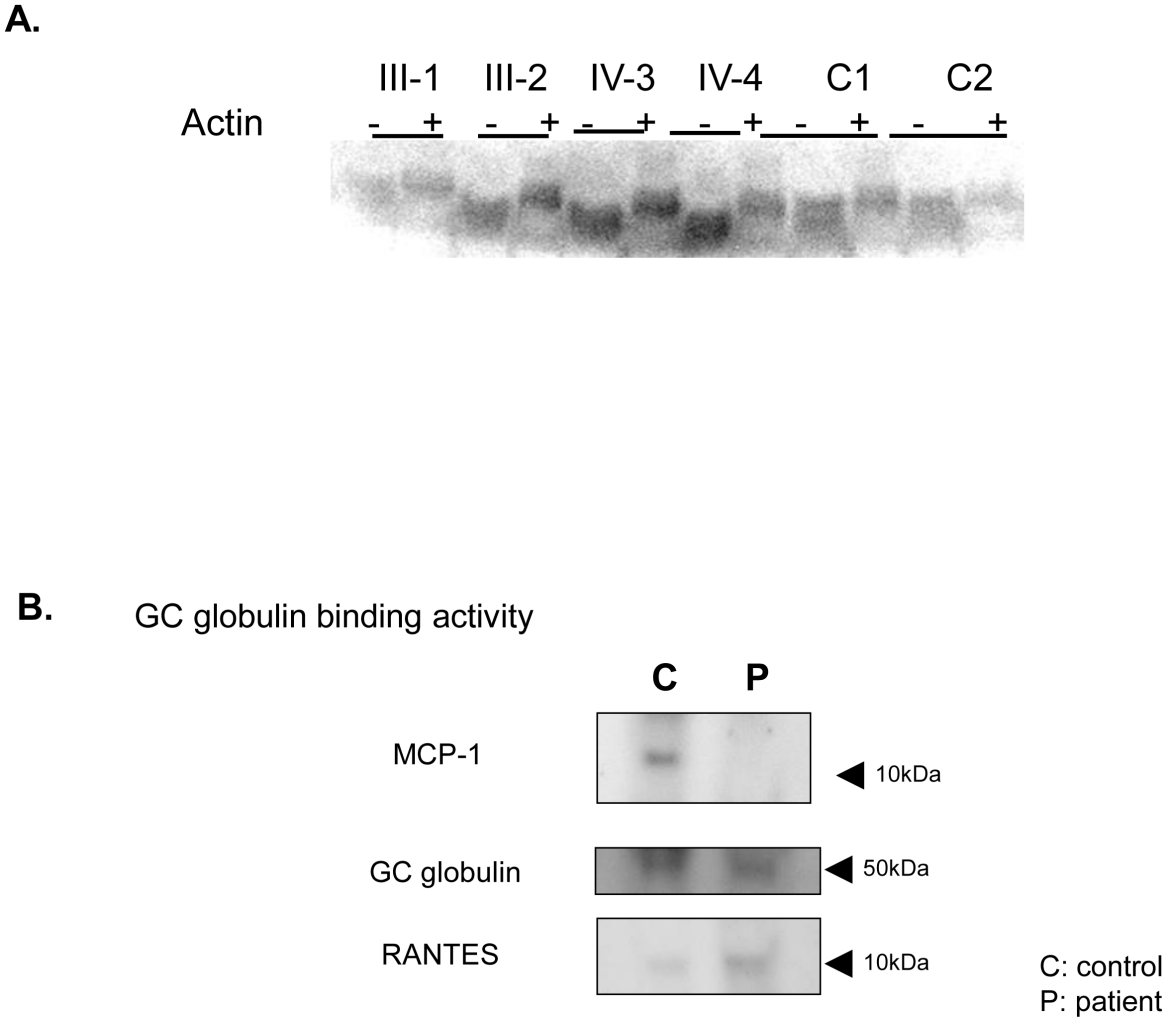
Actin binding activity and immunoprecipitation with GC globulin. **A**) There was no difference in actin binding to GC globulin between the patients and the control subjects. When actin was added to the sample, the molecular weight of GC globulin increased +: sample with actin; -: sample without actin. **B**) HEK 293 cells were transfected with plasmids containing a normal GC globulin sequence allele or a mutant GC globulin sequence allele tagged with GFP. Mutant GC globulin, which had the same mutation as that occurring in the patients, could not bind to MCP-1 but was able to bind to RANTES in the cell culture system.

On the other hand, GC globulin from both the patients and the control subjects bound to actin, as has been previously reported (Figure 3A).

### Dysfunction of GC globulin binding activity to MCP-1 in this pedigree

The binding of MCP-1 to GC globulin in plasma samples from the patients was significantly lower than that in plasma samples from the control subjects; meanwhile, the concentration of serum MCP-1 in the patients tended to be higher than that in the control subjects (Figure 2B). Moreover, the GC globulin variant that was observed in the patients was able to bind to RANTES but not to MCP-1 in the cell culture system (Figure 3B).

## Discussion

We investigated a Japanese family in which some individuals had experienced sudden acute attacks of melalgia accompanied by a migraine and attempted to identify the genetic causality underlying this disorder. Using a linkage analysis, exome re-sequencing, and candidate gene re-sequencing, we identified one novel missense variant in *GC* encoding GC globulin that could play a role in the pathogenesis of melalgia and migraine. The GC globulin variation occurred at R21L, corresponding to the second amino acid of the mature form; this protein is conserved between chickens and humans. The conservation scores for the estimation of disease causality according to PhyloP (0.929373) and GERP (3.57) indicated that the variant was located within a conserved domain. In addition, the estimated effect of the amino acid substitution, as determined using SIFT (0.01) and PolyPhen2 (0.99), suggested that the variant was “probably damaging.”

The group-specific component globulin (GC globulin), also known as vitamin D binding protein (DBP), is a 51–58 kDa multifunctional plasma protein synthesized in large quantities by hepatic parenchymal cells and is secreted into the circulation as a monomeric mature peptide of 458 residues and structural domains.

The function of GC globulin is associated with its role as an actin-scavenging protein in the vascular and extracellular system, working in concert with gelsolin. Moreover, it also plays roles in some non-actin-scavenging functions. GC globulin binds vitamin D and vitamin D analogs with different affinities at a binding site located in the N-terminal region of domain I [18], [19].

Another function of GC globulin involves the release (from activated T and B cells) of enzymes that process the O-linked carbohydrate side chains of GC globulin, transforming this molecule into GC-MAF. In turn, GC-MAF stimulates macrophage activity at the tissue site. GC globulin exerts immunomodulatory functions by enhancing complement factor 5 (C5)-mediated signaling [8].

In neuroinflammatory reactions, such as neuropathic pain, immune cell activity is typically associated with a higher expression of chemokines, such as MCP-1, and cytokines are believed to be key contributors to chronic pain. In the present study, we found that GC globulin can bind to MCP-1 (CCL2) and RANTES in human plasma. Notably, the GC globulin binding affinity to MCP-1 was stronger than that to RANTES.

In the patients in the present study, the GC globulin binding affinity to MCP-1 was lower than that in the control subjects. The GC globulin variant in this family might have affected the binding affinity of GC globulin to MCP-1, possibly because of an alteration in the three-dimensional structure of the GC globulin variant.

MCP-1, a member of the monocyte chemoattractant protein family, and its receptor CCR2 have been implicated in many disorders. With respect to chronic neuroinflammation, a critical role for MCP-1 has been established in animal models for multiple sclerosis, while experimental evidence for acute neuroinflammation has suggested a detrimental role of MCP-1 in stroke and excitotoxic injury [20].

As for the relationship between MCP-1 and neuropathic pain, Franceschini et al. reported that the trigeminal ganglia of a mouse model of familial hemiplegic migraine (R192Q knock in mouse) had higher mRNA and protein levels of MCP-1; they also reported elevated levels of MCP-1 in the plasma of migraine patients [21]–[23]. On the other hand, MCP-1 can also trigger calcitonin gene-related peptide (CGRP) release [24]. In the cranial circulation, CGRP is released by perivascular nerves after trigeminal nerve activation, where it induces cranial vasodilatation by binding to the CGRP receptor [25]. In general, the trigeminal system provides an important pain-transmitting link from the cranial vasculature to the CNS. During migraine attacks, there is a marked increase in the plasma levels of CGRP in the confluence of the cranial venous blood or external jugular vein [26]. However, at the same time, there is no change in the CGRP levels in the peripheral blood [27].

In the present study, we did not observe any difference in the CGRP plasma levels between the patients and the control subjects. However, whether or not the CGRP levels might increase in the confluence of the cranial venous blood or external jugular vein of the patients remains unknown. In conclusion, the dysfunction of GC globulin alters GC globulin-binding activities to cytokines, especially MCP-1. Thus, MCP-1 might play important roles in melalgia and migraine pain.

## Materials and Methods

### Subjects

#### Family

A three-generation Japanese family with 14 members (6 men, 8 women), including 4 patients who had experienced repeated episodes of pain in their extremities, was recruited at Misato Central Hospital, Saitama Prefecture, Japan (see Figure 1A). The proband (V-2), a 45-year-old woman, had experienced repeated episodes of sudden, but transient, pain in her extremities since childhood. Because her mother (IV-1) had also experienced similar episodes, a genetic component was suspected. Consequently, the recruitment of extended family members was performed.

All the affected individuals had experienced repeated episodes of severe pain with an abrupt onset in the extremities, beginning during childhood. The episodes of severe pain lasted for 1–2 hours and did not have any appreciable precipitating factor. In most of the cases, a headache occurred concomitant with the pain. We diagnosed such headaches as migraines in accordance with the International Classification of Headache Disorders (ICHD)-3beta diagnostic criteria [28]. The characteristics of 5 individuals who had experienced migraines are shown in Table 3. The body mass index (BMI) of all the patients was within the normal range.

**Table 3:** Profile of family pedigree.

	Age(years)	Sex	Migraine onset and characteristics	Migraine type
II-3	68	F	Onset at time of first menstruation; simultaneously associated with limb pains	MO
II-5	59	M	No symptoms	
III-2	45	F	Had experienced photophobia and phonophobia prior to onset of migraine attacks at the age of 13 years. Also experienced limb pains. Her migraine headaches were severer during menstruation.	MA
IV-3	12	F	Since childhood; simultaneously associated with limb pains	MO
IV-4	20	F	Since childhood; simultaneously associated with limb pains	MO

Four women with the *GC* gene mutation exhibited repeated episodes of transient, severe myalgia during childhood accompanied by migraines. However, one man with the *GC* gene mutation had never experienced myalgia or a migraine. F: female; M: male; MA: migraine with aura; MO: migraine without aura.

Although we checked the hormone levels, such as estrogen (estragiol [E2]), progesterone, and testosterone, in all the patients, all the values were within the normal ranges. Moreover, the laboratory data, including the calcium and phosphate levels, were within the normal ranges in all the patients.

Genomic DNA was extracted from peripheral blood or saliva samples according to the standard protocols. The ethical committee of the Tokai University School of Medicine and the Chiba University School of Medicine approved the study, and all the participants in the family provided written informed consent.

### Linkage analysis

The 14 subjects (II-1, II-2, II-3, II-4, II-5, III-1, III-2, III-3, III-4, III-5, IV-1, IV-2, IV-3, and IV-4) were genotyped using the Genome-Wide Human SNP Array 5.0 (Affymetrix, Santa Clara, CA, USA); 250 ng of genomic DNA was used for each subject. The SNPs were genotyped with Affymetrix annotation; monomorphic SNPs, X-linked SNPs, and SNPs with Mendelian errors were then excluded. We divided the overall data set into 20 subsets by selecting one every 20 successive SNPs. Then, we performed a multi-point linkage analysis for each of the 20 subsets, and calculated the average LOD scores for all the subsets. Genotype calls with confidence scores higher than 0.95 were treated as “missing”. The LOD scores were calculated using the program MERLIN 1.1.2 [29] under the autosomal-dominant modes of inheritance with reduced penetrance (e.g. 0.99).

### Exome re-sequencing

Equal amounts of DNA from 3 affected individuals in the family (III-1, IV-1, and V-2) were pooled. A total of 3 µg of pooled DNA was subjected to a whole exon capture procedure using the SureSelect Human All Exon Kit (Agilent, Santa Clara, CA, USA), according to the manufacturer's protocols. The captured DNA library was then sequenced using Genome Analyzer IIx (Illumina, San Diego, CA, USA). We used two lanes of paired-end 114-bp reads, which generated approximately 4 Gb of sequence reads. The reads were mapped to the reference genome (UCSC hg18, NCBI build 36.3) using the BWA program (version 0.5.7). The program SAMtools was used to call targeted bases, and differences from the reference sequence were regarded as potential variations. The identified variants were annotated based on the dbSNP build 130 and the CCDS database (version 20090327).

### Filtering of candidate variants

We confirmed the coverage depth and the variant quality and only retained high-quality variants satisfying 3 criteria: 1) the SNP quality had to be no less than 20, 2) no less than 20 reads had to support the variant allele, and 3) the SNP could not be located in a segmental duplicated region (over 0.95 similarity). Variants identified during exome sequencing were then filtered using the following procedures: first, the variants identified during exome re-sequencing were filtered based on the linkage results. Variants located within the linkage regions with LOD scores exceeding 1.50 were selected. Next, we filtered the remaining variants based on an allele frequency of 40%–60% as the dominant model (since affected individuals within a family are expected to share the same causal variant), and we then applied a pooled method. Among these variants, non-synonymous variants, splice-site variants, and short coding insertions or deletions were retained as candidate variants. Finally, all novel variants that were not registered in the dbSNP database were analyzed using the Sanger method on an ABI 3730 (Life Technologies, Carlsbad, USA) to confirm the genotypes of all the family members.

For follow-up confirmation of the co-segregation of the variant with the disease status, we designed PCR primers flanking approximately 300 bp of the variant and sequenced the PCR products from each member of the family using the Sanger method. In addition, 238 control subjects were genotyped to obtain the allelic frequency in the Japanese population.

### Measurement of vitamin D (25 (OH) D, 1,25 (OH)_2_ D), and calcitonin-gene related peptide (CGRP)

We measured the levels of 25 (OH) D, 1,25 (OH)_2_ D, and CGRP in sera from the patients (n = 3) and healthy control subjects (n = 6). Both the 25 (OH) D and the 1, 25 (OH)_2_ D levels were measured using radioimmunoassays. As for the CGRP measurement, the serum samples were extracted using a C-18 reverse phase cartridge with 4% acetic acid and were dried using vacuum centrifugation. Then, the samples were reconstituted with EIA buffer. We measured the prepared samples using an EIA kit (Bertin Pharma, Montigny le Bretonneux, France).

### Profiling of cytokine expression and GC globulin binding activity using protein arrays

Whole blood samples were collected from the patients (n = 3) and the control subjects (n = 6). Plasma was obtained from the whole blood samples by centrifugation. The plasma was then incubated with RayBioTM Human Cytokine Antibody Array 1 (RayBiotech, Norcross, GA, USA) according to the manufacturer's protocol. The resulting data were analyzed using standard densitometry software (Cool saver, Image Gauge; Fuji Photo Film Co., Ltd. and Kohshin Graphic Systems, Inc. Tokyo, Japan).

The results were expressed as the mean ± SD. The statistical analysis was performed using an ANOVA. A value of *P*<0.05 was considered statistically significant.

### Actin binding activity

To evaluate the binding activity to actin, serum samples (40 µL) from each of the patients and control subjects were added to 10 µL of F-actin derived from rabbit skeletal muscle, followed by incubation for 5 minutes at room temperature. After separation using non-denaturing polyacrylamide gel electrophoresis, western blotting was performed using anti-GC globulin antibody raised in rabbits, as described previously [30], [31].

### Immunoprecipitation with GC globulin in sera from patients and control subjects

To evaluate the immunoprecipitation with GC globulin, HEK293 cells were plated in 10-cm dishes and mutant or wild-type pEGFP-GC globulin gene plasmids were transfected into the HEK 293 cells using the lipofectamine method (Trans-IT [Mirus]), according to the manufacturer's instructions. Three days after the transfection, the cells were harvested and homogenized with a cell lysis buffer (50 mM Tris-HCl [pH 7.4], 1% Triton X-100, 0.5 mM PMSF [a protease inhibitor], 2 mM CaCl_2_, and a proteinase inhibitor). The samples were then centrifuged and the resulting supernatant was mixed with anti-GFP-agarose beads at 4°C overnight. The solution was then subjected to immunoprecipitation. Finally, the expressions of MCP-1, GC globulin, and RANTES antibodies were examined using western blot analyses.
